# Addition of high-risk HPV testing improves the current guidelines on follow-up after treatment for cervical intraepithelial neoplasia

**DOI:** 10.1054/bjoc.2000.1689

**Published:** 2001-03

**Authors:** M A E Nobbenhuis, C J L M Meijer, A J C van den Brule, L Rozendaal, F J Voorhorst, E K J Risse, R H M Verheijen, T J M Helmerhorst

**Affiliations:** Department of Obstetrics and Gynaecology, University Hospital Rotterdam, PO Box 2040, CA, 3000, Rotterdam; Department of Pathology; Department of Clinical Epidemiology and Biostatistics; Department of Obstetrics and Gynaecology, University Hospital Vrije Universiteit, PO Box 7057, Amsterdam, MB, 1007, The Netherlands

**Keywords:** human papillomavirus, cervical intraepithelial neoplasia, cervical dysplasia, post-treatment CIN, guidelines

## Abstract

We assessed a possible role for high-risk human papillomavirus (HPV) testing in the policy after treatment for cervical intraepithelial neoplasia (CIN) 2 or 3 (moderate to severe dysplasia). According to the Dutch guidelines follow-up after treatment consists of cervical cytology at 6, 12 and 24 months. Colposcopy is only performed in case of abnormal cervical cytology. In this observational study 184 women treated for CIN 2 or 3 were prospectively monitored by cervical cytology and high-risk HPV testing 3, 6, 9, 12 and 24 months after treatment. Post-treatment CIN 2/3 was present in 29 women (15.8%). A positive high-risk HPV test 6 months after treatment was more predictive for post-treatment CIN 2/3 than abnormal cervical cytology (sensitivity 90% and 62% respectively, with similar specificity). At 6 months the negative predictive value of a high-risk HPV negative, normal smear, was 99%. Largely overlapping, partly different groups of women with post-treatment CIN 2/3 were identified by HPV testing and cervical cytology. Based on these results we advocate to include high-risk HPV testing in monitoring women initially treated for CIN 2/3. In case of a high-risk HPV positive test or abnormal cervical cytology, colposcopy is indicated. All women should be tested at 6 and 24 months after treatment and only referred to the population-based cervical cancer screening programme when the tests are negative on both visits. © 2001 Cancer Research Campaign http://www.bjcancer.com


British Journal of Cancer (2001) **84**(6), 796–801
© 2001 Cancer Research Campaign
doi: 10.1054/ bjoc.2000.1689, available online at http://www.idealibrary.com on IDEAL®

http://www.bjcancer.com

### Addition of high-risk HPV testing improves the current guidelines on follow-up after treatment for cervical intraepithelial neoplasia

**MAE Nobbenhuis[1,2], CJLM Meijer[2], AJC van den Brule[2], L Rozendaal[2], FJ Voorhorst[3], EKJ Risse[2], RHM Verheijen[4] and TJM Helmerhorst[1]**

[1]Department of Obstetrics and Gynaecology, University Hospital Rotterdam, PO Box 2040, 3000 CA, Rotterdam, [2]Department of Pathology, [3]Department of Clinical Epidemiology and Biostatistics, [4]Department of Obstetrics and Gynaecology, University Hospital Vrije Universiteit, PO Box 7057, 1007 MB, Amsterdam, The Netherlands

**Summary** We assessed a possible role for high-risk human papillomavirus (HPV) testing in the policy after treatment for cervical intraepithelial neoplasia (CIN) 2 or 3 (moderate to severe dysplasia). According to the Dutch guidelines follow-up after treatment consists of cervical cytology at 6, 12 and 24 months. Colposcopy is only performed in case of abnormal cervical cytology. In this observational study 184 women treated for CIN 2 or 3 were prospectively monitored by cervical cytology and high-risk HPV testing 3, 6, 9, 12 and 24 months after treatment. Post-treatment CIN 2/3 was present in 29 women (15.8%). A positive high-risk HPV test 6 months after treatment was more predictive for post-treatment CIN 2/3 than abnormal cervical cytology (sensitivity 90% and 62% respectively, with similar specificity). At 6 months the negative predictive value of a high-risk HPV negative, normal smear, was 99%. Largely overlapping, partly different groups of women with post-treatment CIN 2/3 were identified by HPV testing and cervical cytology. Based on these results we advocate to include high-risk HPV testing in monitoring women initially treated for CIN 2/3. In case of a high-risk HPV positive test or abnormal cervical cytology, colposcopy is indicated. All women should be tested at 6 and 24 months after treatment and only referred to the population-based cervical cancer screening programme when the tests are negative on both visits. © 2001 Cancer Research Campaign http://www.bjcancer.com

**Keywords**: human papillomavirus; cervical intraepithelial neoplasia; cervical dysplasia; post-treatment CIN; guidelines

After treatment for high-grade cervical intraepithelial neoplasia (CIN) failure rates of 5–15% have been observed (Gunasekera et al, 1990; Benedet et al, 1992; Alvarez et al, 1994; Mitchell et al, 1998). One of the drawbacks of close cytological follow-up after treatment is that many women present with abnormal cytology but in only about 40–60% of them an underlying CIN lesion is present, indicating high sensitivity but low specificity for post-treatment CIN (Bigrigg et al, 1994; Bollen et al, 1999). Colposcopic examination, as an adjunct to cytology, is often inadequate because of the difficulty in interpreting features of the post-treatment cervix, resulting in unnecessary diagnostic procedures (Bigrigg et al, 1994).

According to the Dutch guidelines, as formulated by the Dutch Society of Cervical Pathology and Colposcopy in 1995, follow-up after treatment for CIN 2 or 3 (moderate to severe dysplasia) consists of cytological follow-up at 6, 12 and 24 months after treatment. Only in the case of an abnormal cervical smear is colposcopic examination indicated (Heintz, 1995; Bollen et al, 1999). After three consecutive negative smears women return to the cervical cancer screening programme. In some other European countries monitoring also consists of cytological follow-up (Duncan, 1992; Chua and Hjerpe, 1997; Mann et al, 1999). For instance, in the UK a total of six smears within 5 years of follow-up are recommended before routine recall. However, in spite of these national guidelines the follow-up policies still vary from centre to centre, indicating a need for evaluation and better implementation.

*Received 18 September 2000*
*Revised 18 December 2000*
*Accepted 19 December 2000*

*Correspondence to:* TJM Helmerhorst; Email: helmerhorst@gyna.azr.nl

It is assumed that effective treatment for CIN lesions results in the eradication of the high-risk human papilloma virus (HPV) infection present before treatment (Elfgren et al, 1996). Persistent infection with high-risk HPV types is required for the development and progression of primary CIN lesions (Remmink et al, 1995; Ho et al, 1998; Nobbenhuis et al, 1999). High-risk HPV is also often present in post-treatment CIN (Chua et al, 1997).

In this observational study we evaluated the rationale for our current follow-up policy, and whether addition of high-risk HPV testing contributes to a better risk-assessment of post-treatment CIN.

### PATIENTS AND METHODS

From 1990–96, 184 women diagnosed with CIN 2 or 3 (moderate and severe dysplasia) at the colposcopy outpatient clinic of the University Hospital Vrije Universiteit in Amsterdam and consecutively treated by cone biopsy or colposcopic guided large loop excision of the transformation zone (LLETZ) were included in this study. All fulfilled the following inclusion criteria: an adequate HPV sample (β-globin PCR-positive) at initial treatment; at least one adequate HPV sample after treatment; no previous history of cervical pathology; no prenatal DES (diethylstilboestrol) exposure; and no concomitant cancer. The median follow-up time was 24 months (range 3–76 months). The study protocol was approved by the ethics review board of the hospital.

### Cervical cytology and HPV testing

In this prospective, observational study post-treatment follow-up was performed by cervical cytology and HPV testing at 3, 6, 9, 12 and 24 months after initial treatment. Since high-risk HPV testing was used for the evaluation of the current follow-up policy, the test results were blinded until the analysis. Cervical scrapes were obtained using a cervex® brush (International Medical Products, Zutphen). After a smear was made on a glass slide the brush was placed in a buffer solution (PBS) and sent to the laboratory for HPV detection (Walboomers et al, 1995).

Cervical smears were classified according to the KOPAC classification, the standard classification in The Netherlands (Hanselaar, 1995). Smears were cytomorphologically classified as Pap 1 (normal), Pap 2 (very mild dyskaryosis), Pap 3a (mild to moderate dyskaryosis), Pap 3b (severe dyskaryosis), Pap 4 (suspected of carcinoma in situ) and Pap 5 (suspected of at least micro-invasive carcinoma). According to the guidelines, colposcopic examination including sampling for histological verification of suspect lesions was only performed in case of a cytomorphologically abnormal smear (≥ Pap 3a, mild dyskaryosis or worse) (Heintz, 1995; Helmerhorst and Wijnen, 1998; Bollen et al, 1999). All histological samples were reviewed by an expert pathologist who was unaware of the clinical findings.

A β-globin PCR was performed to ascertain the quality of the target DNA. HPV testing was performed by EIA PCR using HPV-general-primer-mediated PCR with the general primers GP 5+/6+. All 14 high-risk HPV types (16, 18, 31, 33, 35, 39, 45, 51, 52, 56, 58, 59, 66 and 68) were tested for in one assay. In addition, the PCR amplification products were analysed for individual high-risk HPV types. This test has been described earlier and clinically validated (Remmink et al, 1995; Jacobs et al, 1997; Nobbenhuis et al, 1999).

### Study endpoint

The study endpoint was post-treatment CIN 2/3 defined as a histologically confirmed CIN 2 or 3 lesion after previous treatment. Follow-up ended when patients reached this endpoint. According to the Dutch guidelines women returned to the population-based cervical cancer screening programme after three consecutive negative cervical smears within 24 months after treatment, since these women are considered not to have an elevated risk for post-treatment CIN 2 or 3 (Heintz, 1995; Helmerhorst and Wijnen, 1998).

### Statistical analysis

We used two-by-two tables to assess the diagnostic value for post-treatment CIN 2/3 of a high-risk HPV test and a cervical smear at 3, 6, 9, 12 and 24 months after initial treatment, respectively. In these analyses women without a suspected cervical lesion on colposcopic examination, or with CIN 0 (no CIN) or CIN 1 (mild dysplasia) in the biopsy were considered as 'negative'. For these analyses, the last observations were carried forward for women who had already reached the endpoint and women who returned to their general practitioner before 24 months of follow-up. Women with repeated negative cervical smears were considered to have a colposcopically normal cervix. The McNemar test was used to identify a significant difference in HPV testing and cytology for women with post-treatment CIN 2/3 at different time-points.

**Table 1** Characteristics of the 184 women included in the study

| Characteristic | | Number of patients *n* | (%) |
|---|---|---|---|
| High-risk HPV test at initial treatment | Positive | 181 | (98.4) |
| | Negative | 3 | (1.6) |
| Histology at time of initial treatment | CIN 2 | 9 | (4.9) |
| | CIN 3 | 175 | (95.1) |
| Treatment | LLETZ | 152 | (82.6) |
| | Cone biopsy | 32 | (17.4) |
| High-risk HPV test 3 months after treatment | Positive | 48 | (26.1) |
| | Negative | 136 | (73.9) |
| Cervical smear 3 months after treatment | Abnormal | 31 | (16.8) |
| | Normal | 153 | (83.2) |
| Follow-up | Post-treatment CIN 2/3 | 29 | (15.8) |
| | No evidence of disease | 155 | (84.2) |
| Histology post-treatment CIN 2/3 | CIN 2 | 9 | (31.0) |
| | CIN 3/cancer* | 20 | (69.0) |

*One woman developed cervical cancer after initial treatment for CIN 3.

### RESULTS

### Characteristics of the study group

The mean age at baseline was 34 years (range 21–70 years). Of the included 184 women, 152 were treated by LLETZ and 32 women by cone biopsy (see Table 1). At initial treatment three women (1.6%) with a CIN 3 lesion had negative high-risk HPV tests, both in the cervical smear and biopsy, and remained negative during follow-up after treatment. HPV type 16 was the most prevalent high-risk HPV type at baseline, accounting for 116 of the 181 (64.1%) high-risk HPV-positive women. After treatment, high-risk HPV remained detected in 48 of the 184 women (26.1%). Post-treatment CIN 2/3 was seen in 29 (15.8%) women with a median time until diagnosis of 6 months (range 3–39 months).

### Post-treatment CIN 2/3

The characteristics of the 29 women with post-treatment CIN 2/3 are presented in Table 2. All women with post-treatment CIN 2/3 had CIN 3 at initial treatment and the mean age was 35 years (range 21–58 years). Seventy-two percent (21 of 29) of the cases were diagnosed within 1 year after treatment. Three months after initial treatment the high-risk HPV test was positive in 27 of the 29 cases (93%). The most prevalent high-risk HPV type was HPV type 16, accounting for 81% (22 of 27) of the HPV types. In two women with post-treatment CIN 2/3 no high-risk HPV could be demonstrated in the biopsy or additional treatment tissue. One of them (patient 19) had a high-risk HPV positive test 3 months after treatment and cleared this infection before 6 months of follow-up. In 26 of the 29 (89.7%) women with post-treatment CIN 2/3 the same high-risk HPV type could be detected in the post-treatment lesion as at initial treatment. This could indicate that the treatment did not result in eradication of the virus. Only one woman (patient 21) with an initial HPV type 16 infection cleared this type and acquired HPV type 58, 19 months after treatment. Two women, one with CIN 2 (patient 19) and one with CIN 3 (patient 20), had a high-risk HPV-negative test at post-treatment CIN 2/3.

In another woman, initially treated for a small CIN 3 lesion by LLETZ, follow-up after treatment ended after 28 months because

© 2001 Cancer Research Campaign

**Table 2** Characteristics of patients with post-treatment CIN 2/3 ($n = 29$)

| Patient | Age | Initial treatment | | Test 3 months after treatment | | Follow-up until (months) | | | Post-treatment CIN | |
|---|---|---|---|---|---|---|---|---|---|---|
| | | Treatment | HPV type | Cytology | HPV type | Abnormal cytology* | High-risk HPV* | Post-treatment CIN | Histology | HPV type |
| 1 | 28 | LLETZ | 16/33 | 3b | 16 | pers | pers | 4 | CIN 3 | 16 |
| 2 | 35 | LLETZ | 16 | 3a | 16 | pers | pers | 6 | CIN 3 | 16 |
| 3 | 33 | LLETZ | 16/31 | 3b | 16/31 | pers | pers | 3 | CIN 3 | 16/31 |
| 4 | 29 | LLETZ | 16 | 3a | 16 | pers | pers | 6 | CIN 3 | 16 |
| 5 | 28 | LLETZ | 16 | 3b | 16 | pers | pers | 4 | CIN 3 | 16 |
| 6 | 31 | LLETZ | 58 | 3b | 33/58 | pers | pers | 8 | CIN 2 | 58 |
| 7 | 58 | LLETZ | 33 | 3a | 33 | pers | pers | 5 | CIN 3 | 33/35 |
| 8 | 25 | LLETZ | 16 | 3a | 35 | pers | pers | 5 | CIN 2 | 16 |
| 9 | 36 | LLETZ | 16 | 3b | 16 | pers | pers | 3 | CIN 3 | 16 |
| 10 | 38 | LLETZ | 33 | 3b | 16 | pers | pers | 3 | CIN 3 | 33 |
| 11 | 56 | Cone biopsy | 16 | 4 | 33 | pers | pers | 7 | CIN 3 | 16 |
| 12 | 25 | LLETZ | 16 | 3b | 16 | pers | pers | 3 | CIN 2 | 16 |
| 13 | 21 | LLETZ | 16 | 4 | 16 | pers | pers | 6 | CIN 3 | 16/54 |
| 14 | 51 | LLETZ | 16 | 4 | 16/54 | pers | pers | 3 | CIN 3 | 16 |
| 15 | 27 | LLETZ | 16 | 3a | 16 | pers | pers | 4 | CIN 2 | 16 |
| 16 | 54 | LLETZ | 16 | 3a | 16 | pers | pers | 4 | CIN 2 | 16 |
| 17 | 28 | LLETZ | 16 | 3b | 16 | pers | pers | 3 | CIN 3 | 16 |
| 18 | 23 | Cone biopsy | 16 | 2 | 16 | 7 | pers | 7 | CIN 3 | 16 |
| 19 | 35 | LLETZ | 16 | 1 | 16 | 19 | – | 19 | CIN 2 | – |
| 20 | 27 | LLETZ | 16 | 1 | – | 6 | – | 9 | CIN 3 | – |
| 21 | 35 | LLETZ | 16 | 2 | – | 19 | 19 | 23 | CIN 3 | 58 |
| 22 | 42 | LLETZ | 16 | 1 | 16 | 20 | pers | 24 | CIN 2 | 16 |
| 23 | 33 | LLETZ | 33/35 | 1 | 33/35 | 10 | pers | 10 | CIN 2 | 33/35 |
| 24 | 51 | Cone biopsy | 16 | 1 | 16 | 7 | pers | 7 | CIN 3 | 16 |
| 25 | 33 | Cone biopsy | 16 | 2 | 16 | 22 | pers | 22 | CIN 2 | 16 |
| 26 | 38 | LLETZ | 16 | 1 | 16 | 39 | pers | 39 | CIN 3 | 16 |
| 27 | 34 | LLETZ | 16 | 1 | 16 | 28 | pers | 28 | Cancer | 16 |
| 28 | 31 | LLETZ | 16 | 2 | 16 | 15 | pers | 15 | CIN 3 | 16 |
| 29 | 31 | LLETZ | 16 | 2 | 16 | 15 | pers | 15 | CIN 3 | 16 |

Cytology: Pap 1 = normal dyskaryosis; Pap 2 = very mild dyskaryosis; Pap 3a = mild to moderate dyskaryosis; Pap 3b = severe dyskaryosis; Pap 4 = suspected of carcinoma in situ. *pers = persistent abnormal cervical cytology or high-risk HPV positive after initial treatment (range, time until next visit during follow-up: 2–9 months)

*© 2001 Cancer Research Campaign*

of a cervical smear read as Pap 4 (suspect for carcinoma in situ). Subsequent colposcopy and biopsy showed cervical carcinoma. The intermittent three cervical smears were read as normal. The four high-risk HPV tests before the diagnosis of cervical cancer were persistently positive for HPV type 16. Histology revealed an undifferentiated small cell carcinoma of the cervix and she underwent radical hysterectomy.

### Prediction of post-treatment CIN 2/3

The high-risk HPV test and cervical smear results at different time-points during follow-up of all participating women are shown in Table 3. At the different time-points two subgroups of women were compared, i.e. women who reached post-treatment CIN 2/3 during follow-up and the remaining women. At 3, 6, 9 and 12 months post-treatment more women with post-treatment CIN 2/3 would be identified by high-risk HPV testing than cervical cytology.

The sensitivity for post-treatment CIN 2/3 among women with a high-risk HPV-positive test or an abnormal cervical smear at 3 months after treatment was 93% vs 58%, respectively (at 6 months 90% vs 62%, at 9 months 90% vs 69%, at 12 months 90% vs 72%, and at 24 months 93% vs 93%). Only at 3 and 6 months after treatment was the sensitivity of a high-risk HPV-positive test significantly higher than that of an abnormal cervical smear (McNemar test $P < 0.01$, and $P < 0.05$, respectively). In women without post-treatment CIN 2/3 the number of high-risk HPV-positive tests or abnormal cervical smears at the different time-points was comparable.

The specificity of a positive high-risk HPV test or an abnormal cervical smear at 3 months after treatment was 86% vs 91%, respectively (at 6 months 92% vs 91%, at 9 months 96% vs 92%, at 12 months 96% vs 95%, and at 24 months 99% vs 96%, respectively).

All 21 women with a high-risk HPV-positive test 3 months after treatment without post-treatment CIN 2/3 cleared the HPV infection during follow-up (median 8 months, range 4–18 months). Among them, 16 women with at least three normal cervical smears returned to their general practitioner. In the remaining five women a colposcopically directed biopsy was taken because of an abnormal cervical smear. In two women no CIN was present, three had a CIN 1 lesion (mild dysplasia).

The negative predictive value of a high-risk HPV-negative, cytomorphologically normal, cervical smear was very high. At 3 months after treatment the negative predictive values of a high-risk HPV-negative cytomorphologically normal smear, or either a high-risk HPV-negative smear or a cytomorphologically normal smear were 98%, 98% and 92%, respectively (at 6 months 99%, 98%, and 93%, and at 24 months 100%, 99% and 99% respectively).

### DISCUSSION

Our results show that at 6 months after treatment for high-grade CIN a positive high-risk HPV test is more predictive for post-treatment CIN 2/3 than abnormal cervical cytology. The negative predictive value of a high-risk HPV-negative cytomorphologically normal cervical smear is very high and the presence of high-risk HPV 24 months after treatment is a risk-factor for post-treatment CIN 2/3. Therefore, we consider high-risk HPV testing valuable in the early detection or prediction of post-treatment CIN 2/3.

Three months after treatment only 26% of the women with a high-risk HPV-positive test at baseline still had a positive high-risk HPV test, indicating that in most women treatment resulted in eradication of high-risk HPV. Cervical cytology was abnormal in 17% of the women, but it is known that reading cervical smears 3 months after ablative treatment is difficult because of the ‘repair-effect’ (Maclean, 1984).

The reason why some women present with post-treatment CIN while the majority do not is unclear. Possible explanations include incomplete removal of the CIN lesion, development of a new CIN lesion by reinfection with HPV, and even the revival of so-called dormant or occult HPV infections (Bistoletti et al, 1988; Nuovo and Pedemonte, 1990). In 90% (26 of 29) of all cases with post-treatment CIN 2/3 we found the same high-risk HPV type as before the initial treatment. This high number agrees with other studies (Chua et al, 1997). Since our HPV assay does not differentiate between HPV type variants we cannot exclude a role for HPV type variants in the genesis of post-treatment CIN 2/3.

At 24 months of follow-up after treatment two out of the 155 (1.3%) women who did not develop post-treatment CIN 2/3 had a positive high-risk HPV test with normal cytology. Since they both had at least three normal cervical smears around the time of acquisition of high-risk HPV they were regarded as having no high-grade CIN lesion and were referred to their general practitioner for screening according to the population-based screening programme. So far, no recurrent CIN disease has been reported in these women.

The relation between a persistent high-risk HPV infection and the development and maintenance of CIN lesions has already been established (Ho et al, 1998; Nobbenhuis et al, 1999). Yet, in two women with post-treatment CIN 2/3 no high-risk HPV type could be found in the CIN lesion or corresponding smear (Table 2). HPV negativity was confirmed by type-specific PCR. The occurrence of high-risk HPV-negative scrapes in cases with cervical dysplasia is in agreement with an earlier study (Nobbenhuis et al, 1999).

Three facts argue for our view of using high-risk HPV testing, next to cervical cytology, in the follow-up after initial treatment for high-grade CIN lesions: the higher sensitivity of a high-risk HPV-positive test than of an abnormal cervical smear, with similar specificity; the high negative predictive value of a high-risk HPV-negative, cytomorphologically normal cervical smear, and, largely overlapping, partly different groups of women with post-treatment CIN 2/3 were identified by HPV testing and cervical cytology. One woman with cervical cancer and another with CIN 3 identified at 28 and 39 months after initial treatment, respectively, had normal cervical smears during follow-up. They would not have been at risk of undue referral to a low-risk group and follow-up procedure if high-risk HPV testing was used to monitor the initial treatment, since all intermittent high-risk HPV tests were positive. In these patients, all cervical smears were revised by an expert panel and were again read as normal.

We advocate to monitor women 6 months after initial treatment both by high-risk HPV testing and cervical cytology. In case of a positive test, colposcopically directed biopsies are indicated. Retesting by both tests should be considered at 24 months after initial treatment to avoid missing cervical carcinomas because of detection problems. Moreover, it is known that acquisition of HPV is increased in women with a history of CIN lesions (Nobbenhuis et al, 1999). Only when cytological and HPV testing are negative during at least 24 months should women be referred to the population-based cervical cancer screening programme. These recommendations will be tested, together with a cost–benefit analysis, in a prospective study involving women treated for high-grade CIN.

© 2001 Cancer Research Campaign

**Table 3** High-risk HPV test and cervical cytology results at 3, 6, 9, 12 and 24 months of follow-up in 184 women initially treated for CIN 2 or 3

| | At 3 months follow-up | | At 6 months follow-up | | At 9 months follow-up | | At 12 months follow-up | | At 24 months follow-up | |
|---|---|---|---|---|---|---|---|---|---|---|
| Post-treatment CIN 2/3* | Yes | No | Yes | No | Yes | No | Yes | No | Yes | No |
| Number | 29 | 155 | 29 | 155 | 29 | 155 | 29 | 155 | 29 | 155 |
| Mild dyskaryosis or worse | 17 | 14 | 18 | 14 | 20 | 13 | 21 | 8 | 27 | 6 |
| Normal cytology | 12 | 141 | 11 | 141 | 9 | 142 | 8 | 147 | 2 | 149 |
| High-risk HPV-positive | 27 | 21 | 26 | 13 | 26 | 6 | 26 | 6 | 27 | 2 |
| and mild dyskaryosis or worse | 17 | 6 | 17 | 7 | 19 | 2 | 20 | 3 | 25 | – |
| and normal cytology | 10 | 15 | 9 | 6 | 7 | 4 | 6 | 3 | 2 | 2 |
| High-risk HPV-negative | 2 | 134 | 3 | 142 | 3 | 149 | 3 | 149 | 2 | 153 |
| and mild dyskaryosis or worse | – | 8 | 1 | 7 | 1 | 11 | 1 | 5 | 2 | 6 |
| and normal cytology | 2 | 126 | 2 | 135 | 2 | 138 | 2 | 144 | – | 147 |

*The last observations are carried forward for women who reached the endpoint and women who returned to their general practitioner before 24 months of follow-up. McNemar test to identify difference in HPV testing and cervical cytology in predicting post-treatment CIN 2/3 at different time points: t3, t6, t9, t12 and t24 was 8.1 ($P < 0.01$), 4.9 ($P < 0.05$), 3.1, 2.3 and 0.3, respectively.

### ACKNOWLEDGEMENTS

We thank Ans Remmink MD and Katja Gaarenstroom MD for collecting data and Kirsten Comes and René Pol for excellent technical assistance.

### REFERENCES

Alvarez RD, Helm CW, Edwards RP, Naumann RW, Patridge EE, Shingleton HM, McGee JA, Hall J, Higgins RV and Malone JM (1994) Prospective randomized trial of LLETZ versus laser ablation in patients with cervical intraepithelial neoplasia. *Gynecol Oncol* **52**: 175–179

Benedet JL, Miller DM and Nickerson KG (1992) Results of conservative management of cervical intraepithelial neoplasia. *Obstet Gynecol* **79**: 105–110

Bigrigg A, Haffenden DK, Sheehan AL, Codling BW and Read MD (1994) Efficacy and safety of large-loop excision of the transformation zone. *Lancet* **343**: 32–34

Bistoletti P, Zellbi A, Moreno-Lopez J and Hjerpe A (1988) Genital papillomavirus infection after treatment for cervical intraepithelial neoplasia (CIN) III. *Cancer* **62**: 2056–2059

Bollen LJM, Tjong-A-Hung SP, Mol BW, van der Velden J, ten Kate FJW, ter Schegget J and Bleker OP (1999) Prediction of recurrent cervical dysplasia by human papillomavirus detection among patients with abnormal cytology. *Gynecol Oncol* **72**: 199–201

Chua KL and Hjerpe A (1997) Human papillomavirus analysis as a prognostic marker following conization of the cervix uteri. *Gynecol Oncol* **66**: 108–113

Duncan ID (1997) *Guidelines for Clinical Practice and Programme Management*, 2nd edn, pp 13–14. National Coordinating Network, NHS Cervical Screening Programme, London

Elfgren K, Bistoletti P, Dillner L, Walboomers JMM, Meijer CJLM and Dillner J (1996) Conization for cervical intraepithelial neoplasia is followed by disappearance of human papillomavirus deoxyribonucleic acid and a decline in serum and cervical mucus antibodies against human papillomavirus antigens. *Am J Obstet Gynecol* **174**: 937–942

Gunasekera PC, Phipps JH and Lewis BV (1990) Large loop excision of the transformation zone (LLETZ) compared to carbon dioxide laser in the treatment of CIN: a superior mode of treatment. *Br J Obstet Gynaecol* **97**: 995–998

Hanselaar AGJM (1995) Het bevolkingsonderzoek op baarmoederhalskanker. Een uniform model voor cytopathologisch onderzoek. *Med Contact* **50**: 1590–1592

Heintz APM (1995). Het bevolkingsonderzoek op baarmoederhalskanker. Patiënten met een afwijkende uitstrijk. Vervolg-en natraject. *Med Contact* **50**: 1593

Helmerhorst ThJM and Wijnen JA (1998) Richtlijnen bevolkingsonderzoek baarmoederhalskanker. *Ned Tijdschr Obstet Gynaecol* **111**: 264–265

Ho GYF, Bierman R, Beardsley L, Chang CJ and Burk RD (1998) Natural history of cervicovaginal papillomavirus infection in young women. *N Engl Med J* **338**: 423–428

Jacobs MV, van den Brule AJC, Snijders PJF, Meijer CLJM, Helmerhorst ThJM and Walboomers JMM (1997) A general primer GP5+/6+ mediated PCR-EIA for rapid detection of high- and low-risk HPV DNA in cervical scrapes. *J Clin Microbiol* **35**: 791–795

Maclean AB (1984) Healing of cervical epithelium changes after laser treatment of cervical intraepithelial neoplasia. *Br J Obstet Gynaecol* **91**: 697–706

Mann CH, Kehoe S, Brown A and Luesley DM (1999) A study of the follow up patterns of women treated for CIN 2 and 3 before and after the introduction of the 1992 guidelines. *Br J Obstet Gynaecol* **106**: 1126–1129

Mitchell MF, Tortolero-Luna G, Cook E, Whittaker L, Rhodes-Morris H and Silva E (1998) A randomized clinical trial of cryotherapy, laser vaporization, and loop electrosurgical excision for treatment of squamous intraepithelial lesions of the cervix. *Obstet Gynecol* **92**: 737–744

Nobbenhuis MAE, Walboomers JMM, Helmerhorst ThJM, Rozendaal L, Remmink AJ, Risse EKJ, van der Linden HC, Voorhorst FJ, Kenemans P and Meijer CJLM (1999) Relation of human papillomavirus status to cervical lesions and consequences for cervical-cancer screening. *Lancet* **354**: 20–25

Nuovo GJ and Pedemonte BM (1990) Human papillomavirus types and recurrent cervical warts. *JAMA* **263**: 1223–1226

Remmink AJ, Walboomers JMM, Helmerhorst ThJM, Voorhorst FJ, Rozendaal L, Risse EKJ, Meijer CJLM and Kenemans P (1995) The presence of persistent

*© 2001 Cancer Research Campaign*

high-risk HPV genotypes in dysplastic cervical lesions is associated with progressive disease: natural history up to 36 months. *Int J Cancer* **61**: 306–311
Walboomers JMM, Jacobs MV, van Oostveen JW, van den Brule AJC, Snijders PJF and Meijer CJLM (1995) Detection of genital human papillomavirus infections and possible clinical implications. In: *Human Papillomavirus Infections in Dermatovenerology*, Gross G and Von Krogh G (eds), pp 341–364. CRC Press, New York

© 2001 Cancer Research Campaign
